# Rosemarinic Acid-Induced Destabilization of Aβ Peptides: Insights from Molecular Dynamics Simulations

**DOI:** 10.3390/foods13244170

**Published:** 2024-12-23

**Authors:** Liang Zhao, Weiye Jiang, Zehui Zhu, Fei Pan, Xin Xing, Feng Zhou, Lei Zhao

**Affiliations:** 1 Key Laboratory of Geriatric Nutrition and Health, Beijing Technology and Business University, Ministry of Education, Beijing 100048, China; liangzhao@btbu.edu.cn (L.Z.); jiangwyjy@163.com (W.J.); zhuzehui0614@163.com (Z.Z.); 2 Beijing Engineering and Technology Research Center of Food Additives, Beijing Technology and Business University, Beijing 100048, China; 3 Institute of Apicultural Research, Chinese Academy of Agricultural Sciences, Beijing 100093, China; yunitcon@yeah.net; 4 Beijing Key Laboratory of Functional Food from Plant Resources, College of Food Science and Nutritional Engineering, China Agricultural University, Beijing 100083, China; xingxin@cau.edu.cn

**Keywords:** Alzheimer’s disease, rosmarinic acid, amyloid-β peptide, molecular dynamics, free energy landscape, Poisson–Boltzmann surface area mechanics, neuroprotective

## Abstract

Alzheimer’s disease (AD) is a neurodegenerative disorder marked by the progressive accumulation of amyloid-β (Aβ) plaques and tau protein tangles in the brain. These pathological aggregates interfere with neuronal function, leading to the disruption of cognitive processes, particularly memory. The deposition of Aβ forms senile plaques, while tau protein, in its hyperphosphorylated state, forms neurofibrillary tangles, both of which contribute to the underlying neurodegeneration observed in AD. Rosmarinic acid (RosA), a natural compound found in plants such as *Rosmarinus officinalis*, is known for its antioxidant, anti-inflammatory, and antimicrobial properties. Due to its ability to cross the blood–brain barrier, RosA holds promise as a nutritional supplement that may support brain health. In this study, molecular dynamics (MD) simulations were used to investigate the impact of RosA on the structural stability of Aβ peptides. The results indicated that the addition of RosA increased the instability of Aβ, as evidenced by an increase in the Root Mean Square Deviation (RMSD), a decrease in the Radius of Gyration (Rg), and an expansion of the Solvent Accessible Surface Area (SASA). This destabilization is primarily attributed to the disruption of native hydrogen bonds and hydrophobic interactions in the presence of two RosA molecules. The free energy landscape (FEL) analysis and MM-PBSA (Poisson-Boltzmann Surface Area Mechanics) results further support the notion that RosA can effectively bind to the hydrophobic pocket of the protein, highlighting its potential as a nutritional component that may contribute to maintaining brain health and function.

## 1. Introduction

Alzheimer’s disease (AD) is a progressively worsening neurodegenerative disorder associated with aging, manifesting as a gradual cognitive dysfunction, including a decline in memory and progressive impairments in reasoning, language, and daily living skills [1]. Amyloid protein accumulation and the emergence of intracellular neurofibrillary tangles are pathological symptoms of AD [2]. According to data from the World Health Organization, dementia has become the seventh leading cause of death worldwide [3]. The disease ranks as the fourth leading cause of death among the elderly, following heart disease, cancer, and cardiovascular diseases, and its medical expenses have become a significant economic burden for families and society [4]. Moreover, the number of AD patients in China has increased dramatically, and, globally, over 45 million people are currently affected. It is projected that by 2050, this number will reach 131 million [5]. Therefore, research into the treatment of AD is urgent and critical.

Extracellular amyloid plaques and intracellular neurofibrillary tangles represent the defining pathological features of AD, both of which play crucial roles in the disease’s progression and associated cognitive decline [6]. The main component of senile plaques is an aggregated peptide known as the amyloid-β (Aβ) peptide. According to the amyloid cascade hypothesis, an increase in Aβ accumulation or aggregation initially leads to the formation of Aβ oligomers, followed by fibrils and ultimately plaques [4]. Aβ is a 39–43 amino acid peptide derived from the cleavage of amyloid precursor protein (APP) by β-secretase and γ-secretase, consisting of a predominantly hydrophilic N-terminal domain (1–16) and a C-terminal hydrophobic structural domain [7]. Despite evidence that Aβ monomers may have neuroprotective functions in the brain [8], they are critical for the formation of toxic oligomers, intermediate protofibrils, and mature fibrils [9].

The current drugs used for the treatment of AD are not only limited in number but also often associated with side effects, such as donepezil and rivastigmine, which cannot meet the rapidly increasing demands of AD patients [4]. As societal standards of living enhance and our comprehension of AD evolves, there has been a remarkable renaissance of interest in natural product research over the last decade, and the results are quite interesting [10]. The consumption of natural substances that can improve AD is undoubtedly an important direction and approach for the prevention and treatment of AD. Many studies have demonstrated that oxidative damage is closely associated with the hallmark pathologies of AD [11,12,13]. Many antioxidant compounds, such as vitamin E (DL-α-tocopherol) [14], vitamin A [15], quercetin [16], and nicotine [17] have been suggested to reduce oxidative stress associated with AD. Phenolic compounds, including resveratrol (RES), kaempferol (KAE), and (-)-epigallocatechin gallate (EGCG), have been validated through cell-based assays [18], demonstrating their ability to modify the structure of apoE4 and mitigate its pathogenic role in Alzheimer’s disease (AD). Additionally, olive polyphenolics, such as oleuropein and its derivatives, have been shown to inhibit the aggregation of α-synuclein (αSN), which is involved in neurodegeneration in Parkinson’s disease (PD) [19]. Polar phenolic compounds have also demonstrated the potential to cross the blood–brain barrier in in vitro studies, suggesting their possible neuroprotective effects during the onset or progression of AD.

Rosmarinic acid (RosA), a water-soluble natural phenolic compound, is widely found in plants from various families, including Lamiaceae, Boraginaceae, Cucurbitaceae, Tiliaceae, and Apiaceae [20]. RosA is used to improve health because of its nutritional properties and has been noted to have potent antioxidant activity [21,22]. RosA, a potent polyphenolic antioxidant, along with its derivatives lithospermic acid, rabdossin, and salvialonic acid, exhibits significant bioactivity. These compounds have demonstrated potential in modulating key pathways involved in various diseases, including cancer, diabetes, neurodegenerative disorders, cardiovascular conditions, and inflammation [23]. Especially, RosA has been shown through both in vitro and in vivo animal studies to exhibit significant neuroprotective effects [24]. These cumulative results have spurred investigations into the potential of RosA to destabilize Aβ peptide, aiming to uncover novel therapeutic approaches for AD.

In AD research, pinpointing therapeutic agents is challenging due to the variability in animal models and human dietary influences. Silico modeling provides a powerful simulation tool to more rapidly and fully understand the characteristics of a complex biological system [25]. These silico technologies can accurately predict the tertiary structure of proteins and their interactions with ligands, providing insights into binding sites and the stability of protein–ligand complexes [26]. Such methods offer cost-effective benefits and conserve valuable time and resources [27]. This study investigated the stabilizing effects of RosA, a representative natural polyphenolic molecule, on Aβ peptides using molecular docking, molecular dynamics (MD), and MM-PBSA methods. We initially determined the optimal binding site of RosA with hexameric Aβ peptides (PDBID: 2NAO) through molecular docking and subsequently conducted a 500 ns MD simulation based on this information. Assessing parameters such as the Root Mean Square Deviation (RMSD), Radius of Gyration (Rg), Solvent Accessible Surface Area (SASA), secondary structure, and free energy landscape (FEL), alongside analyzing intermolecular forces and conformational dynamics, revealed RosA’s pivotal role in the destabilization of Aβ fibrils. The results collectively confirmed the destabilizing effect of RosA on Aβ peptides and its preventive potential, establishing a foundation for the use of RosA (and other polyphenolic compounds) as nutritional supplements to support brain health and function.

## 2. Materials and Methods

The initial structure of Aβ peptide required for this study was obtained from the RCSB Protein Data Bank (http://www.rcsb.org/pdb, ID: 2NAO, accessed 23 September 2023), and the three-dimensional structure of RosA (CID: 5281792) was sourced from PubChem (https://pubchem.ncbi.nlm.nih.gov/, accessed 23 September 2023).

### 2.1. Molecular Docking Analysis

Molecular docking simulations were employed to better understand the interactions between the most effective binding sites of RosA and 2NAO. In the docking experiments, the structure of the small molecule ligand was optimized using the Avogadro software package 1.2.0 (Avogadro Development Team, San Diego, CA, USA), with the force field set to MMFF94s to minimize ligand energy and subsequently merge nonpolar hydrogens [28]. The receptor preparation process involved the following steps: (1) removal of water molecules and ligands from the protein structure; (2) verification of the protein for missing atoms, bonds, and contact points using Swiss-PdbViewer 4.10 (Swiss Institute of Bioinformatics, Geneva, Switzerland); (3) addition of polar hydrogen atoms and Kollman charges via AutoDockTools 1.5.6 (TSRI, San Diego, CA, USA); (4) and docking box design using the getbox plugin (https://github.com/MengwuXiao/Getbox-PyMOL-Plugin, accessed 23 September 2023), with coordinates for RosA_1_ (x = 36.0, y = 62.0, z = 56.0) and RosA_2_ (x = 26.0, y = 26.0, z = 32.0), ensuring coverage of all active site residues. Ligand docking was performed with AutoDock Vina 1.1.2 (The Scripps Research Institute, La Jolla, CA, USA), selecting the optimal conformation based on binding affinity (kcal/mol). Final analysis and visualization were conducted using PyMOL 2.5 (Schrödinger, LLC, New York, NY, USA) and Discovery Studio 19.1.0 (BIOVIA, Dassault Systèmes, San Diego, CA, USA) to visualize the docked conformations and analyze the intermolecular interaction forces, highlighting the forces between them [29].

### 2.2. MD Analysis

Given that molecular docking captures a static binding conformation, potentially leading to uncertainties in subsequent analyses, MD simulations are essential to validate and further evaluate the stability of the protein–ligand complex as per the docking outcomes. In this study, GROMACS software package 22.5 (The GROMACS Development Team, Uppsala, Sweden) was used to perform a continuous dynamic analysis of the interaction patterns between RosA and Aβ peptide over a period of 500 ns from both kinetic and thermodynamic perspectives. The optimal binding structures of RosA and Aβ peptide, as determined post-molecular docking, were selected for the simulation. The GROMACS simulation utilized the AMBER14sb force field under explicit solvation conditions. Geometric optimization of RosA was performed using the GAFF force field to correct any unreasonable bond lengths and angles. AmberTools was then employed to generate the GAFF-based topology parameters for the molecules.

All systems were placed in cubic TIP3P water boxes (dimension set at 10 nm × 10 nm × 10 nm), and Na+ and Cl− ions were added to neutralize the total charge of the system, thereby maintaining neutrality. Energy minimization was initially performed using the steepest descent method (1000.0 kJ/mol/nm) [30], followed by a second round of energy minimization using the conjugate gradient optimization method (100.0 kJ/mol/nm). Subsequently, the system was pre-equilibrated using NVT (1 ns, 298 K) and NPT (1 ns, 1 Bar) simulations. Following this, all systems underwent a production simulation of 500 ns. The Particle Mesh Ewald (PME) cut-off was set at 1.0. All observed metrics were calculated using GROMACS analysis tools. PyMOL 2.5.0 and VMD 1.9.4a55 (Theoretical and Computational Biophysics Group, University of Illinois at Urbana-Champaign, Urbana, IL, USA) were used for visualization purposes.

### 2.3. Analytical Methods of Silico Simulation

The GROMACS analysis suite was employed to examine MD trajectories, with the conformations visualized using PyMOL. Specifically, the GROMACS toolkit included tools such as gmx rmsd, gmx gyrate, gmx sasa, and gmx rmsf to evaluate the conformational stability of the system. The dictionary of secondary structure of proteins (DSSP) program was employed to evaluate the secondary structure component of Aβ peptide in the absence and presence of RosA using gmx do_dssp tool. The RMSD is a pivotal parameter that quantifies the fluctuations in the selected elements and serves to delineate the stability of the complex within the protein–small molecule interaction [31]. Rg of the main chain atoms represents the distribution of atoms in a given space relative to their center of mass [32]. This parameter serves not only as an indicator of protein stability during the simulation but also reflects the compactness of the conformation. SASA, which represents the portion of a protein’s surface available for interaction with solvent molecules via van der Waals forces, was determined for the main chain atoms. RMSF values depict the fluctuation levels of protein residues before and after RosA binding, serving as key indicators of receptor structural flexibility during simulation [33].

### 2.4. MM-PBSA Analysis

The calculation of binding free energy through the MMPBSA is a pivotal step in evaluating the structural and functional attributes of molecular complexes, subsequent to MD simulations [34]. Specifically, a trajectory file comprising 500 frames was selected for the calculation of the MM-PBSA binding free energy, and the binding free energy for the complex of RosA with the Aβ peptide was determined utilizing the MM-PBSA approach [35]. The binding free energy of the protein–ligand complex was determined using g_mmpbsa:

ΔG_bind_ = ΔG_complex_ − (ΔG_protein_ − ΔG_ligand_) = ΔG_gas_ + ΔG_sol_ (1)

ΔG_gas_ = ΔG_ele_ + ΔG_vdw_; ΔG_sol_ = ΔG_pol_ + ΔG_(non-pol)_ (2)

Here, ΔG_bind_, ΔG_complex_, ΔG_protein_, and ΔG_ligand_ represent the binding free energy [36], the free energy of the protein–ligand complex, the total energy of the protein in solvent, and the total energy of the ligand in solvent, respectively [37]. The binding affinity of the complex was calculated over the entire 500 ns with a total of 50,000 frames. Following Kumari et al. [35], the binding free energy per residue of the Aβ peptide–RosA complex was calculated to assess the contribution of individual Aβ residues to complex formation; the binding free energy per residue of the protein–ligand complex was measured to ascertain the role of individual residues of Aβ peptide in the Aβ peptide–RosA complex.

### 2.5. Free Energy Landscape (FEL)

In molecular dynamics simulations, the free energy landscape (FEL) is utilized to illustrate the distribution of free energy across different conformations, offering crucial insights into the stability and conformational changes in the molecule. Conformational data derived from MD trajectories are examined, with key collective variables (such as RMSF and Radius of Gyration) selected to represent these variations. GROMACS tools, particularly gmx sham and gmx trjconv, are employed to process the simulation data and identify representative structures. Finally, FEL is visualized in two- and three-dimensional plots, revealing stable conformations and energy minima [34].

## 3. Results and Discussions

### 3.1. Molecular Interaction and Binding Mode

**Figure 1:**
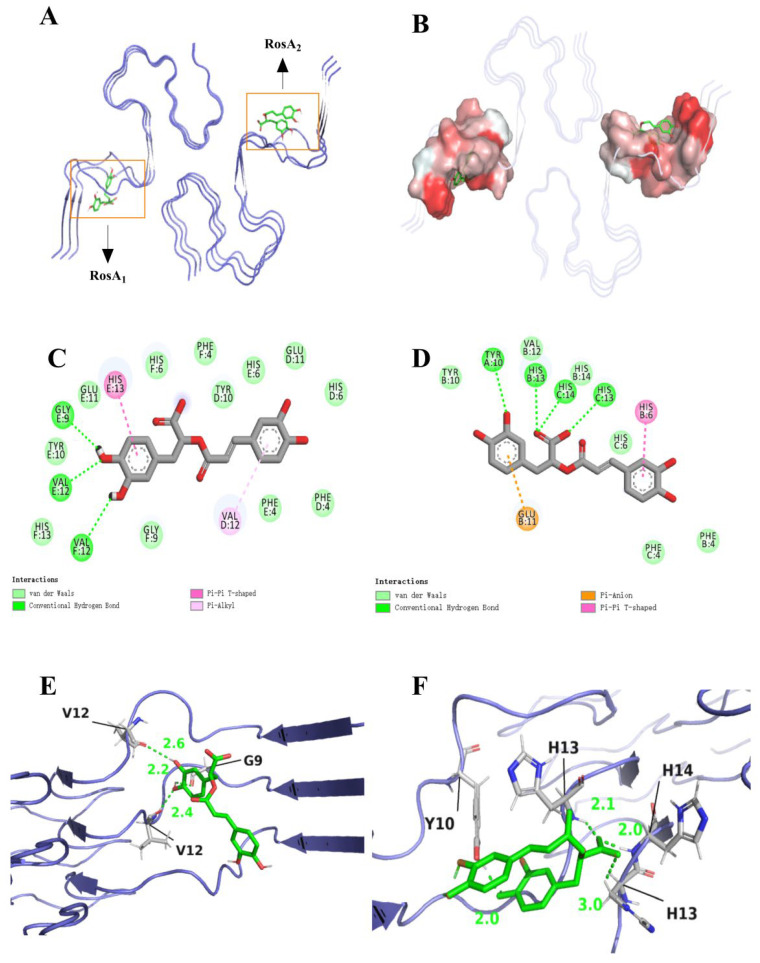
Molecular docking analysis: best-chosen conformation of the binding sites. (**A**) Stabilized 3D conformation of RosA bound to Aβ peptide; (**B**) hydrophobic pocket between RosA and Aβ peptide binding sites; (**C**) 2D view of the interaction of RosA_1_ and (**D**) RosA_2_ binding sites; (**E**) 3D view of RosA_1_; and (**F**) the binding site interactions of the RosA_2_ peptide are shown, with the ligand depicted in gray and hydrogen bonds to the protein residues highlighted in green.

We have delved into the intricate binding affinities between Aβ peptide and two RosA, significantly overcoming the limitation of previous studies where a single ligand could only interact with amyloid monomers [38,39]. The results of our study assessed the binding interactions between RosA and the Aβ peptide. The docking results highlighted key intermolecular forces, including hydrogen bonding, van der Waals interactions, and charge attractions. As illustrated in Figure 1, RosA_1_ binds to the hydrophobic pocket of Aβ through hydrogen bonds with GLY9, VAL12, and VAL12. Consistent with the findings of Ono et al. [40], the interactions, particularly with GLY9, are beneficial for inhibitory activity by engaging small (local) amino acid regions, thereby preventing the aggregation of α-synuclein [41] and Aβ peptide. Additionally, HIS13 forms Pi-Pi T-shaped interactions that are beneficial for binding [41], while VAL12 participates in Pi–Alkyl interactions. RosA_2_ interacts with the hydrophobic pocket via hydrogen bonds with HIS13, HIS14, and TYR10. Pi-Pi T-shaped interactions are also formed with HIS6. The carboxyl group (-COOH) of Glu11 often interacts with the pi bond of RosA_2_, thereby inhibiting the aggregation of the Aβ peptide [42]. Hydrogen bonds can increase the stability of APP–ligand complexes, which is crucial for regulating APP processing and inhibiting the production of Aβ peptides. Carotenoids could hinder the aggregation and fibrillation of Aβ peptides through hydrogen bonds. Van der Waals forces enhanced the stability of these complexes, which is essential for neuroprotection and aids the crossing of the blood–brain barrier by carotenoids, enabling them to exert their effects within the brain [43]. The augmentation of hydrogen bonding and van der Waals interaction sites subsequent to engagement with the Aβ peptide explains that the molecule has a good interaction with the receptor [44]. RosA_1_ selected the best binding conformation from 20 docking poses, with a binding energy of −7.363 kcal/mol, while RosA_2_ chose the optimal binding conformation from 20 different energy conformations, with a binding energy of −6.231 kcal/mol. Bhattacharya et al. [45] found that the binding affinity of BACE1 (β-secretase 1) with Fluspirilene was −9.2 kcal/mol. Similarly, in the study by Shahwan et al. [46], they selected a specific conformation of Donepezil, which exhibited significant affinity for the Aβ peptide, with a binding affinity of −8.1 kcal/mol. The binding energies of these two ligands with the Aβ peptide, as reported in this article, are lower than those observed in other studies, suggesting that they could serve as optimal structural for further investigation.

### 3.2. Trajectory Processing and Inspection

**Figure 2:**
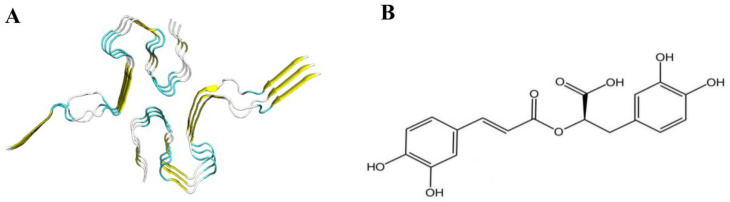
(**A**) Cartoon representation of Aβ peptide; (**B**) the 2D chemical structure of RosA.

The Aβ peptide manifests as a dimeric LS-shaped amyloid, characterized by a dual horseshoe-like configuration, as illustrated in Figure 2A. Each monomeric unit consists of three strands, thus forming a hexameric amyloid protein comprising six strands: A, B, C, D, E, and F. Through the use of PyMol (Vision: 2.5.0), it can be clearly observed that all six strands are full-length residues from 1 to 42. According to reports [47], residues 1 through 14 demonstrate a partially ordered configuration, adopting a β-sheet conformation. Conversely, residues 15 to 42 exhibit a notably compact arrangement, coalescing into a double-horseshoe structure that is characterized by deeply embedded residues and hydrophobic side chains. Notably, each constituent strand in this structure reveals distinct cross-β segments, which constitute essential prerequisites for both the initiation of amyloid protein fibrillation and the maintenance of structural stability.

RosA, as illustrated in Figure 2B, possesses catechol hydroxyl groups within its structure, serving as the foundation for its free radical scavenging activity. The conjugated double bond at the C3 position exerts a synergistic effect, and the number of phenolic hydroxyl groups and potential hydrogen bond-forming groups in the molecule are positively correlated with the molecule’s antioxidant activity, which is an important factor contributing to the strong antioxidant activity of RosA [48].

**Figure 3:**
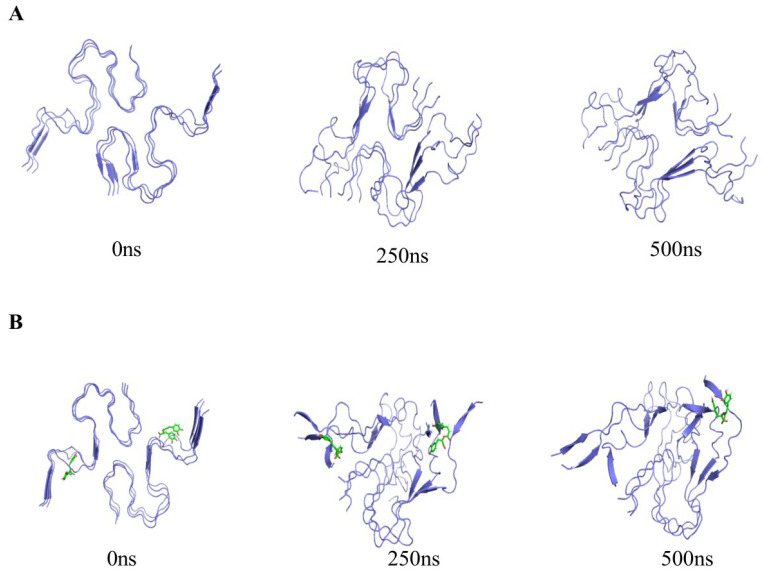
(**A**) Molecular trajectories of Aβ peptide systems during 500 ns MD simulations; (**B**) molecular trajectories of the Aβ-RosA systems during 500 ns MD simulations.

To further understand the aggregation behavior of Aβ amyloid protein and RosA in a realistic solution system, we conducted MD simulations to mimic the environment. Initially, the morphology of Aβ amyloid protein over the course of 0–500 ns was observed in Figure 3A. The Aβ amyloid protein’s structural progression at 0, 250, and 500 ns was meticulously tracked, revealing a clear sequence of events. At the initial 0 ns, the six protein chains were notably flat and free from any significant entanglement. As time progressed to 250 ns, a transformation was evident, with all six chains undergoing bending and beginning to intertwine. By the 500 ns mark, the chains had further evolved into a more tightly woven configuration. The observation that the chains twisted without unfolding suggests that the secondary structure of the protein has been preserved, maintaining a stable fibrillar conformation. This finding is consistent with previous studies on the Aβ amyloid protein that have highlighted the enhanced stability of the amyloid fibrils [49]. The current observations corroborate these reports, reinforcing the understanding of the protein’s aggregation behavior and its structural integrity throughout the simulation period. Figure 3B elucidates the interaction dynamics within the combined system of Aβ amyloid protein and RosA molecules. It is evident that at the 250 ns mark, two RosA molecules have already formed distinct bonds with separate monomer units of the protein, while the six protein chains remain distinguishable. As the simulation progresses to 500 ns, one RosA molecule is observed to maintain its binding to a monomer unit, whereas the other has dissociated from its binding site. In the presence of RosA molecules, the Aβ amyloid protein exhibits a notably disordered and unstable state, as illustrated in Figure 3B. This heightened perturbation and distortion of the Aβ amyloid protein’s structure, compared to its natural hexameric form, suggests an increased level of instability. These findings underscore the potential of RosA as a promising bioactive agent, offering new avenues for the treatment of AD by modulating the aggregation behavior of the Aβ amyloid protein.

### 3.3. Stability Analysis

**Figure 4:**
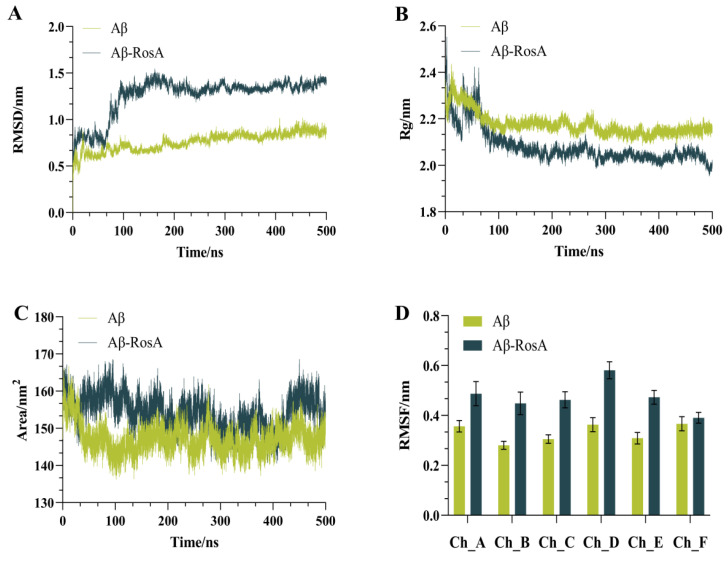
Basic results of MD simulation based on the molecular docking structure. (**A**) The RMSD values of systems; (**B**) the Rg values of systems; (**C**) the SASA values of systems; (**D**) the RMSF values of systems. The horizontal axes labeled Ch_A, Ch_B, Ch_C, Ch_D, Ch_E, and Ch_F correspond to the individual chains A through F of the Aβ peptide.

The MD simulation results have shed light on the significant impact of RosA on the structural stability and aggregation tendency of the Aβ peptide. The RMSD (root-mean-square deviation) values serve as a metric for the deviation from the initial structure, with higher values indicating greater structural changes. Conversely, a lower RMSD signifies minimal deviation, suggesting a more stable conformation. It is generally accepted that an RMSD value below 2 Å denotes a reliable simulation method [50]. As depicted in Figure 4A, all RMSD values are below the 2 Å threshold, demonstrating the reliability of our simulation approach [51]. Notably, the RMSD of the Aβ-RosA complex began to increase markedly after approximately 70 ns, reaching a peak around 150 ns, and then stabilizing at a value of approximately 1.26 nm around 200 ns. In stark contrast, the Aβ system devoid of RosA exhibited a more stable configuration, with RMSD values consistently lower, averaging around 0.75 nm. This contrast indicates that the presence of RosA may reduce the structural stability of Aβ, potentially inducing a transition from a compact to a more disordered state, as corroborated by the referenced study [52]. This effect could be instrumental in impeding the aggregation of the protein, offering valuable insight into the role of RosA in modulating the behavior of Aβ peptide.

Figure 4B provides a detailed account of the conformational effects of RosA on the Aβ peptide, as reflected by the Rg data. At the outset, around 30 ns, the Rg value for the Aβ-RosA complex underwent a brief surge, only to be succeeded by a significant decline that persisted up to 500 ns. A lower Rg value is indicative of a more compact protein structure, implying a higher degree of folding and reduced exposure to the solvent [53]. This trend stands in stark contrast to the Aβ system in the absence of RosA, which displayed higher and more stable Rg values. This suggests that the binding of RosA to the Aβ peptide triggers conformational shifts that lead to a more condensed structure. However, this compactness comes at the cost of stability, rendering the complex more prone to structural disintegration. The interplay between compactness and stability, as illustrated by the Rg values, offers a nuanced perspective on the influence of RosA on the Aβ peptide’s conformation and its potential implications for aggregation and stability.

As the concentration of proteins increases, the SASA of a protein tends to decrease, which can be used to predict changes in protein conformation [54]. The correlation between SASA values and observed conformational changes is clearly depicted in Figure 4C. Notably, the Aβ-RosA complex reached a peak SASA value near 95 ns, after which it gradually declined toward a stable state. This suggests an initial expansion of the receptor’s hydrophobic pocket upon binding with RosA, followed by a stabilization of the protein structure. Moreover, the Aβ-RosA complex consistently showed higher SASA values compared to the unbound Aβ, indicating that the binding of RosA significantly reduces the protein’s propensity to aggregate. This effect is crucial in significantly reducing the abnormal clustering of Aβ peptide.

Figure 4D showed RMSF curves across six different trajectories, including both the control and RosA-treated samples. The findings indicated that the RMSF values for all six chains within the Aβ-RosA complex were higher than those observed for Aβ in isolation, signifying an increase in chain mobility following RosA binding. This heightened flexibility, supported by numerous studies, along with a substantial alteration in structure, implies that RosA may induce a destabilization of the Aβ complex [55,56].

The MD simulations reveal that RosA significantly perturbs Aβ structure, as indicated by elevated RMSD and altered Rg and SASA parameters. This destabilization is likely due to the disruption of Aβ’s native hydrogen bonding and hydrophobic interactions by RosA, consistent with its observed anti-amyloidogenic effects in vitro [57]. Moreover, it has been reported that RosA could inhibit both oligomerization and Aβ deposition in AD transgenic mice (Tg2576) [58]. The introduction of RosA significantly modified the structural properties of the Aβ protein, decreasing its stability and potentially preventing the formation of pathological aggregates by facilitating the protein’s unfolding. These discoveries contribute valuable insights for the advancement of innovative therapeutic approaches aimed at addressing AD.

### 3.4. Secondary Structure in the Presence of RosA

The temporal evolution of the secondary structure for both the Aβ peptide and the Aβ-RosA complex was delineated using the DSSP program, offering a comparative graphical representation of their structural profiles.

**Figure 5:**
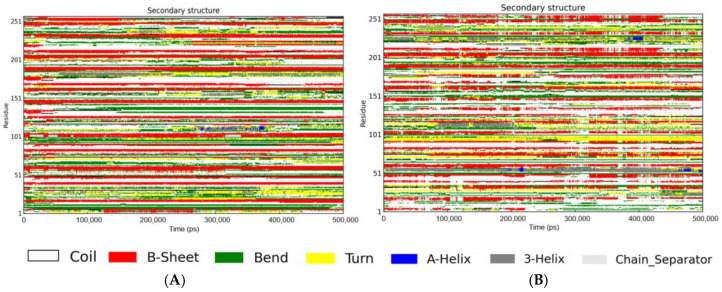
DSSP-based secondary structure time evolution over 500 ns. (**A**) The secondary structure time evolution of Aβ peptide; (**B**) the secondary structure time evolution of Aβ-RosA system.

As depicted in Figure 5A and Table 1, the secondary structure analysis revealed that the Aβ peptide in isolation exhibited a composition of 1% helix, 27% β-sheet, 42% coil, 21% bend, and 7% turn conformations. In contrast, Figure 5B illustrates the secondary structure of the Aβ-RosA complex, which displayed a composition of 3% helix, 22% β-sheet, 45% coil, 18% bend, and 9% turn conformations. The interaction between RosA and the Aβ peptide led to an increase in coil content from 42% to 45% and a concomitant decrease in β-sheet structure from 27% to 22%. The findings indicate a destabilization of the Aβ peptide’s ordered β-sheet structure upon binding with RosA. This observation is corroborated by the work of Singh et al., who reported that the addition of rk10 to Aβ protofibrils resulted in an increase in coil content and a reduction in β-sheet content, indicative of the destabilization of the Aβ protofibril structure by rk10 [59]. Furthermore, these results are consistent with the research by Dong et al., where hesperetin was found to significantly diminish the β-sheet conformational transition in proteins, underscoring its potential as a therapeutic agent for AD [39].

### 3.5. MMPBSA Binding Free Energy Analysis

**Figure 6:**
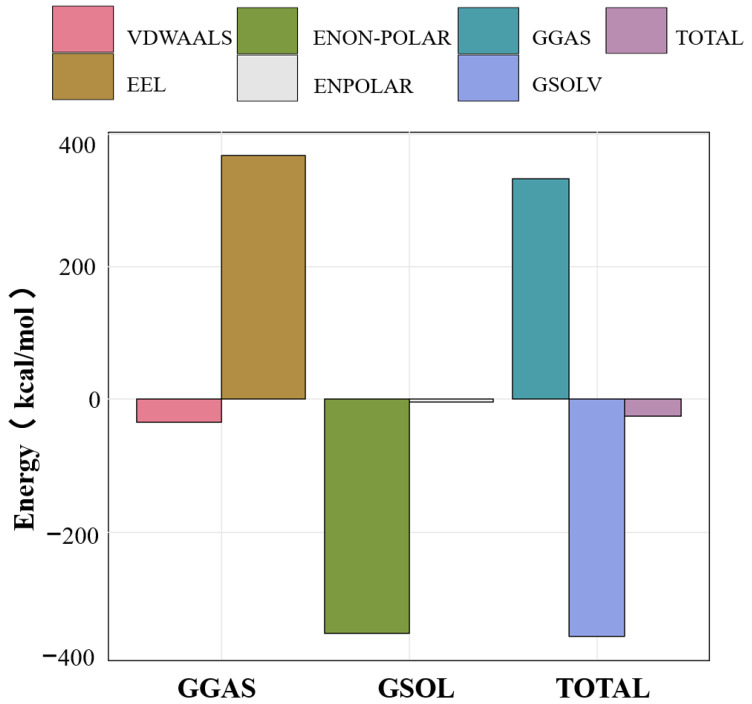
Binding energy between RosA and Aβ peptide calculated using MM-PBSA, along with the breakdown of individual energy components. All free energy values are given in units of kcal/mol.

To investigate the changes and differences in binding energy between RosA and the Aβ peptide during the dynamic process, the binding energy of RosA and the protein was examined. The results for the different energy contributions of RosA and the Aβ peptide complex are shown in Figure 6. This included the examination of van der Waals forces (VDWAALS) and electrostatic energy (EEL) in both the gas phase (GGAS) and solvent phase (GSOLV), as well as their sums (TOTAL).

The calculated values for ΔE_vdw_, ΔG_poland_, ΔG_non-pol_, and ΔG_sol_ were all negative, signifying that van der Waals forces, polar solvation free energy, nonpolar solvation energy, and solvation free energy all contribute positively to the binding process. Notably, the polar and nonpolar solvation free energies play a predominant role in this interaction. ΔG_pol_, representing the polar solvation energy, shows a positive contribution to the energy, which is counterproductive to the system’s stability. However, this effect promotes the spontaneous binding of RosA to the Aβ peptide. The negative binding free energy indicates that the association of ligands and receptors is a spontaneous process that can achieve equilibrium, with lower energy correlating to greater system stability [54].

The MMPBSA energy calculations disclosed that the interaction between RosA and the Aβ peptide is thermodynamically stable, evidenced by a negative binding free energy (ΔG_bind_) of −26 kcal/mol. This negative ΔG_bind_ is indicative of a spontaneous binding process, as documented in this study [60], and suggests the formation of a robust and stable RosA-Aβ complex. Among the various components of the binding energy, only the electrostatic component (ΔG_eel_), with a contribution of 367 kcal/mol, is unfavorable to the binding. The exergonic nature of the interaction implies a high binding affinity and a reduced likelihood of complex dissociation, which are favorable characteristics for RosA’s potential as a therapeutic agent against Aβ proteins. In conclusion, the calculations highlight the favorable thermodynamic profile of the RosA-Aβ interaction, underscoring its potential therapeutic application.

### 3.6. Conformational Changes and Force Analysis

Expanding on the insights gained from MD simulations, we delved into the intricate interactions between RosA and the protein, transcending the constraints imposed by the static outcomes of molecular docking studies. The minimal energy area, highlighted in red on the free energy contour map, is indicative of the complex’s enhanced stability. The smaller and more centrally located blue regions further suggest that the corresponding complexes exhibit greater stability [61]. This visual representation from the contour map provides a nuanced understanding of the binding dynamics, reinforcing the stability of the RosA–protein complex.

**Figure 7:**
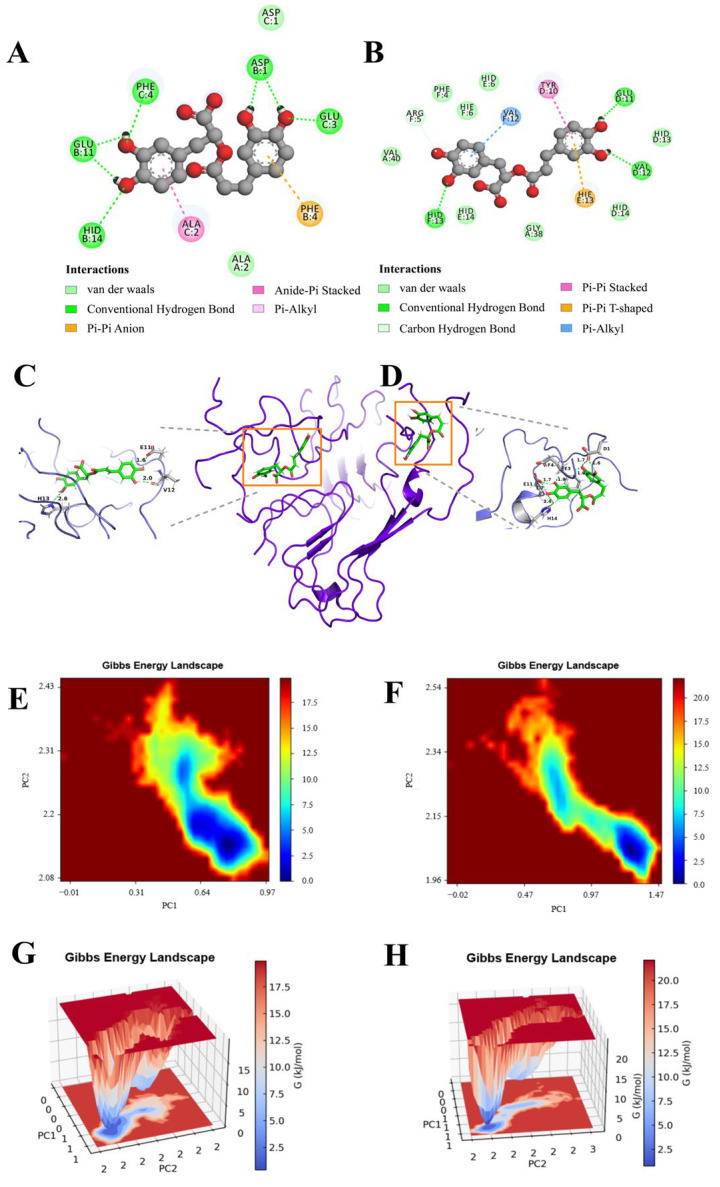
FEL results. (**A**) The lowest energy conformation diagram of the Aβ protein–RosA_1_; (**B**) the lowest energy conformation diagram of the Aβ peptide–RosA_2_; (**C**) 3D view of RosA_1_; (**D**) RosA_2_ protein binding site interactions, where the ligand is indicated in gray and the hydrogen bonding with the protein amino acid is indicated in green; (**E**) 2D free energy landscape of RosA_1_; (**F**) 2D free energy landscape of RosA_2_; (**G**) 3D free energy landscape of RosA_1_; (**H**) 3D free energy landscape of RosA_2_.

The FEL analysis has revealed that the dynamics of RosA within the Aβ peptide–RosA complex underwent notable changes during the simulation phase, diverging from the static perspectives offered by molecular docking. The observed reduction in the size of the energy well implies a relative decrease in the system’s stability. In terms of the nature of the interactions, RosA_1_ established a greater number of hydrogen bonds with pivotal Aβ protein residues, such as HID13, VAL12, and GLU11. Additionally, it engaged in Pi–Alkyl interactions with VAL12, Pi-Pi Stacked interactions with TYR10, and Pi-Pi T-shaped interactions with HIE13 (Figure 7A). Similarly, RosA_2_ formed an increased number of hydrogen bonds with key Aβ peptide residues, including PHE4, GLU11, HID14, GLU3, and ASP1 (Figure 7B). It also participated in Pi-Pi Stacked interactions with PHE4 and Amide-Pi Stacked interactions with ALA2. These detailed interactions underscore the complex and dynamic nature of the Aβ-RosA binding, highlighting the multifaceted role of hydrogen bonds and various types of non-covalent interactions in stabilizing the complex.

The FEL (Figure 7E,F) and MM-PBSA (Figure 7G,H) analyses confirm the thermodynamic stability of the RosA-Aβ complex, characterized by a negative binding free energy. This suggests that RosA can effectively compete with Aβ’s self-assembly into toxic aggregates. Notably, our results show a decrease in Aβ’s β-sheet content upon RosA binding, which is crucial since β-sheets are central to Aβ toxicity [62]. This observation is in line with studies demonstrating that small molecule incorporation can destabilize Aβ protofibrils [38]. The comprehensive analysis of these interactions provides a deeper understanding of the molecular basis for the binding affinity and potential therapeutic efficacy of RosA in the context of Aβ peptide interactions.

## 4. Conclusions

The study presented herein delves into the influence of rosmarinic acid (RosA) on Aβ aggregation, a pivotal process in Alzheimer’s disease (AD). Through molecular docking, dynamics simulations, and free energy calculations, we demonstrate that RosA exerts a significant destabilizing effect on the Aβ peptide. Our findings also indicate a significant decline in the β-sheet composition of Aβ following RosA binding, a critical element in ameliorating its neurotoxic properties. Noteworthy is the strong binding affinity between RosA and Aβ, as manifested by the disruption of the peptide’s native conformation and the manifestation of a negative binding free energy, highlighting the establishment of a stable complex. This investigation underscores the potential health benefits of RosA in AD and calls for further investigation into its viability as a natural adjunct for neurodegenerative disorders.

Building on our results, we plan to further explore the binding mechanisms of RosA with various structural forms of Aβ aggregates. We also intend to incorporate an experimental validation of small molecule interactions and expand our analysis by including additional PDB structures. This comparative approach will enhance the breadth and significance of our research. Additionally, we aim to investigate the effect of RosA on the conformational changes in amyloid fibrils, with a particular focus on their dynamic behavior in solution. By increasing the number of RosA, we seek to understand its role in regulating the movement and stability of Aβ aggregates, providing deeper insights into the potential of RosA as a therapeutic agent for Alzheimer’s disease.

## Figures and Tables

**Table 1:** The secondary structure contents of the simulated systems.

Secondary Structure Component	Aβ Peptide	Aβ Peptide System
^a^ Helix	1	3
^b^ β-Sheet	27	22
Coil	42	45
Bend	21	18
Turn	7	9
Chain_separator	2	2

^a^ Helix = α-helix + 3-helix. ^b^ β-sheet = β-strand + β-bridge.

## Data Availability

The original contributions presented in the study are included in the article; further inquiries can be directed to the corresponding authors.
